# ARoCuS Web application promotes standardized treatment and documentation of rotator cuff tears

**DOI:** 10.1007/s12306-020-00658-8

**Published:** 2020-04-20

**Authors:** S. G. Walter, D. Cucchi, W. Thomas, M. J. Friedrich, T. Jansen

**Affiliations:** 1 Department for Orthopedic Surgery, University Hospital, Siegmund-Freud-Str. 25, 53127 Bonn, Germany; 2 Clinic for Orthopedic Surgery, Karol Wojtyla Hospital, Viale Africa 32, 00144 Rome, Italy

**Keywords:** Rotator cuff tear, Classification, ARoCuS, Rotator cuff reconstruction, Shoulder, Arthroscopy

## Abstract

**Purpose:**

To program a Web application for simplified calculation of the Advanced Rotator Cuff tear Score (ARoCuS), which is a 5-part, 18-item treatment-oriented intraoperative scoring system for intraoperative evaluation of rotator cuff tears.

**Methods:**

ARoCuS characteristics (torn tendon, tear size, tissue quality and tear pattern) were assessed intraoperatively on 40 consecutive patients with rotator cuff tears for calculation of defect category Δ*V*. Video recordings were used to re-calculate the ARoCuS after surgery and to assess inter-observer reliability.

**Results:**

The Web application “ARoCuS App” was built using Angular and transformed to a native iOS application. The intraoperative use of the app proved to be simple and intuitive. There were inter-/intra-observer differences neither in ARoCuS defect categories Δ*V* nor in ARoCuS characteristics (*p* > 0.05).

**Conclusion:**

The ARoCuS app is a supportive tool for integration of standardized treatment procedures and documentation of rotator cuff tears in clinical routine.

## Introduction

Rotator cuff tears are one of the most frequent pathologies of the shoulder, with an estimated prevalence of 16.9–24.5% among the general population (asymptomatic and symptomatic) [1, 2], and will therefore certainly remain within the focus of orthopedic research. Surgical repair of rotator cuff (RC) tears is a successful keystone in orthopedic surgery, with the number of publications regarding surgical techniques and results increasing steadily every year [3]. Nowadays, arthroscopic techniques are considered as gold standard to surgically address most RC tears as they showed similar functional results to open and mini-open surgery in the short- and mid-term follow-up and a decreased rate of postoperative complications [4].

Numerous techniques have been described to address rotator cuff lesions, with the aim of providing a mechanically stable repair at the tendon-to-bone junction. However, not every rotator cuff tear can be treated by the same surgical technique [5]. Thus, multiple classifications are available to categorize rotator cuff tears of the shoulder, each of which considers different characteristics, such as the quality of the muscle of the torn tendon, size and shape of the tear, degree of retraction and fatty infiltration [6–10]. As tear size and complexity increase, it becomes more difficult to provide a comprehensive and standardized description by a single classification. This makes the comparison of rotator cuff tears troublesome when judged by different surgeons using different classification systems. The Advanced Rotator Cuff tear Score (ARoCuS) is an intraoperative classification tool, which integrates crucial structural characteristics of a rotator cuff tear and defines different defect severity categories. ARoCuS is a 5-part, 18-item scoring system leading to an overall ARoCuS value that can be categorized into four categories Δ*V* (ARoCuS grade) with increasing tear’s extent. Thus, the main advantage of the ARoCuS is the possibility to integrate macroscopic characteristics of the torn tendon and its grade of mobilization into a dynamic classification system, which can be predictive for repair success and can thus guide the surgeon to the best choice among the numerous options available [11].

So far, the score’s usage in daily clinical routine has been limited due to its awkward calculation. Therefore, a Web tool has been programmed to enhance practicability and promote a wider standardized treatment as well as comparability of clinical studies reporting on rotator cuff tears.

This study was designed to evaluate whether the ARoCuS Web application is suitable for intraoperative as well as video-based evaluation of rotator cuff tears according to the ARoCuS classification and to test the Web application for inter- and intra-observer reliability.

## Methods

### Study design

In the time between 2017 and 2018, all patients that were referred to our institution to undergo arthroscopic rotator cuff reconstruction due to symptomatic rotator cuff tears were assessed for eligibility. Exclusion criteria were age less than 18 years, previous surgery to the index shoulder and presence of unequivocally diagnosed concomitant disorders of the shoulder, including shoulder stiffness, fracture, osteonecrosis or infection. Inclusion criteria were the presence of symptomatic rotator cuff tears, confirmed by MRI findings, with a fatty infiltration of the supraspinatus tendon of grade ≤ 2 according to the Fuchs classification [12].

#### Preoperative investigation

Prior to surgery, all patients underwent a standardized clinical examination in which the UCLA score was assessed [13]. The RC tear was confirmed by MRI imaging in all patients.

### Surgical technique

Surgery was performed in general anesthesia and additional brachial plexus anesthesia with the patient in beach chair position. Each step of the rotator cuff evaluation during the diagnostic arthroscopy was documented by photographic and video recordings to allow for postoperative re-assessment.

A three-portal arthroscopic technique was used. The size and number of anchors to be used were determined intraoperatively according to ARoCuS, based on the size and pattern of the tear, as well as the size of the footprint and bone stock. After the tendon had been mobilized, the footprint was prepared and the anchors were inserted. The sutures were then passed through the rotator cuff in a configuration that is determined by the tear pattern and repaired to the footprint. In most of the cases, a double-row configuration was conducted. In patients with type 2 or 3 acromial morphology, according to Bigliani’s classification a subacromial decompression was performed before RC repair [14].

All the patients were operated by a single surgeon (M.F.). The patients were discharged 2–3 days after operation wearing a sling to limit abduction and internal or external rotation (Ultrasling III; DonJoy, Carlsbad, CA, USA). The patients were allowed to start active physical therapy to regain muscle strength 8 weeks after surgery.

#### Postoperative evaluation

Routine clinical follow-ups were scheduled 6 weeks and 6 months postoperatively. During the clinical evaluation, the UCLA score was recorded, as well as information on patient’s satisfaction and postoperative complications.

### ARoCuS classification

The ARoCuS classification assesses anatomic *characteristics* (and ranked, specific (sub-)items) of RC tears:

1. *Torn muscle/tendon (M)* including the following sub-items: supraspinatus muscle, subscapular muscle, infraspinatus muscle and teres minor muscle.
2. *Tear size (S)* differentiated into: small tears < 1 cm, medium tears 1–3 cm and large tears > 3 cm.
3. *Tissue quality of the torn tendon (T)* with sharp, clear edges, some fraying or severe fraying.
4. *Pattern of the tear (P)* including the following sub-items: articular-sided partial thickness tears, bursal-sided partial thickness tears, crescent-shaped tears, L- or T-shaped tears and massive/complex tears.
5. In cases of massive and complex tears, the extent of *mobilization (mob)* of the torn tendon is described by one of the following sub-items: mobilization, reduced mobilization and immobilization.

Based on these characteristics, the severity of the tear/defect category Δ*V* is calculated by applying the following equation:

$$\Delta V = (0.56 \cdot M \cdot S + 1.02 \cdot M \cdot T){\text{PMOB}} - 3.00$$

Initially, Δ*V* and rotator cuff tear characteristics were documented manually. For simplification, an app was developed.

### App design

**Fig. 1:**
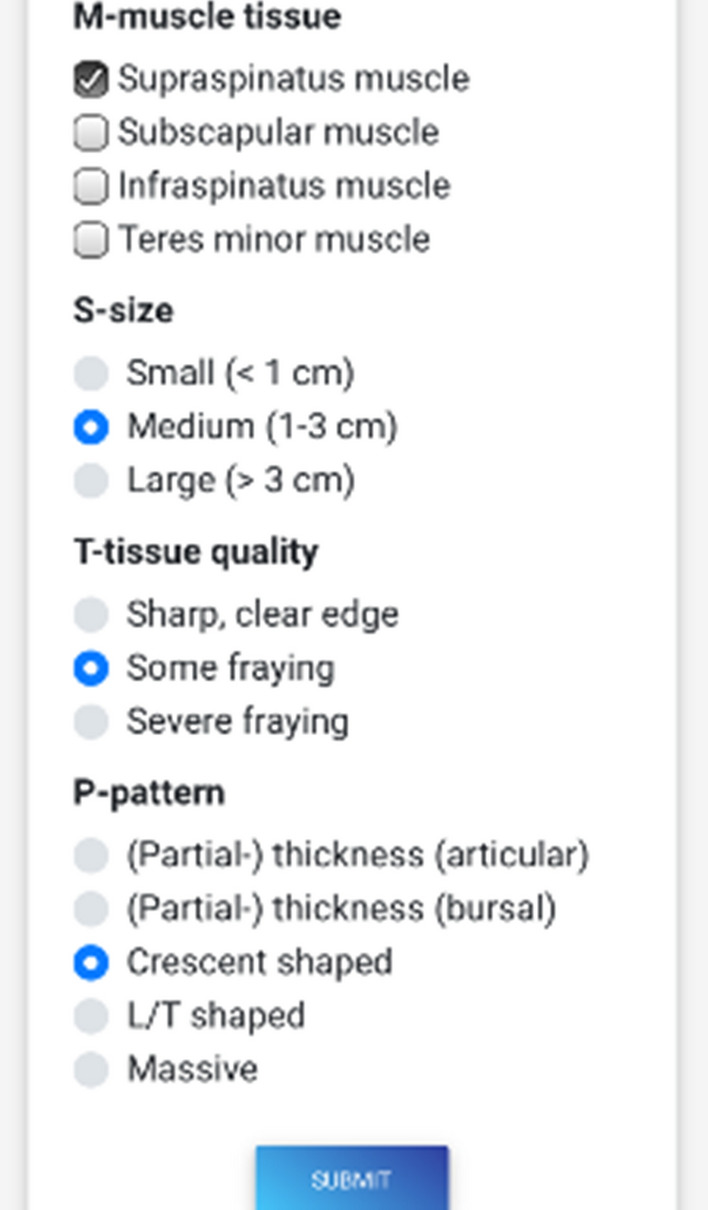
Screenshot of the ARoCuS application’s surface. For each element of the ARoCuS dimensions (size, tissue quality, pattern and mobilization), one choice can be made. For the element muscle (*M*), several choices can be made. In this case, the chosen tendon ranked the highest is used for calculation of Δ*V*

The Web application “ARoCuS App” was built using Angular (Version 6), a TypeScript-based open-source Web application framework. The front-end design was built using Bootstrap. Cordova Apache was used to transform the Web app to a native iOS application. All data are temporarily stored in the internal storage of the device and are deleted after each run time. No data are stored on the Web server (Fig. 1).

The app itself is designed as drop-down menu. Each sub-item of the tear characteristics can be chosen by a simple bullet button. Once a sub-item for each characteristic is chosen, the defect category Δ*V* can be calculated. Additionally, the tears dimensions are displayed in the MSTPmob format for reproduction and documentation.

### Inter- and intra-observer reliability of the ARoCuS classification

Intraoperatively, the surgeon evaluated the rotator cuff tear by means of the ARoCuS classification. Data on tear characteristics were inserted directly into the ARoCuS app by an assistant in order to simultaneously calculate the resulting defect categories.

Subsequently, tears were treated according to the calculated defect category Δ*V*. Based on intraoperative photographs and video documentation, tear dimensions were re-evaluated by a second orthopedic surgeon, blinded to the patient’s history and the surgical procedure for evaluation of inter-observer reliability. At minimum 6 months after the initial rating, the orthopedic surgeon evaluating the rotator cuff tear’s characteristics based on video material repeated the evaluation for assessment of intra-observer reliability.

### Time for calculation of Δ*V* with and without ARoCuS app

Based on video recordings of the last consecutive ten patients, time for calculation of Δ*V* without (usage of a standard pocket calculator without formula function) and with ARoCuS app was determined. Time was measured from the start of the calculation until the result of Δ*V*. Initial miscalculations were counted.

### Statistics

All statistical analyses were performed using SPSS 24.0 (IBM, Chicago, IL). Continuous variables were tested for normal distribution and eventually expressed as the mean ± standard deviation (SD) or medians and first and third quartiles [Q1–Q3] as appropriate, depending on the characteristics of data distribution. For inter-observer reliability of the total ARoCuS defect category Δ*V* and for continuous variables, interclass coefficients (ICCs) were calculated. Inter-observer reliability of the individual categorical features was determined using kappa statistics. For all analyses, the significance level was set at *p* value lower than 0.05.

## Results

Forty consecutive patients were prospectively enrolled. Demographic data of the study population are reported in Table 1. All ARoCuS Δ*V* defect categories were observed and rated by the surgeon. There were 27 small defects (Δ*V* I), 8 medium defects (Δ*V* II), 3 large-sized defects (Δ*V* III) and 2 massive rotator cuff tears (Δ*V* IV; Table 2).

**Table 1:** Basic demographic data of the study population

Demographics	Data
Age in years	58.4 ± 8.7
Right/left	25/15
Male/female	18/22

**Table 2:** Overview of rotator cuff tear properties and the resulting defect categories of forty consecutive cases rated by two orthopedic surgeons (OS 1 and 2)

	*M* (muscle)				*S* (size)			*T* (tissue quality)			*P* (pattern)					Mob (mobilization)			Δ*V*			
	SSP	SC	IS	TM	I	II	III	I	II	III	I	II	III	IV	V	I	II	III	I	II	III	IV
OS 1	38	4	2	1	6	25	9	10	22	8	3	3	25	7	2	5	2	2	27	8	3	2
OS 2	38	4	2	1	6	25	9	9	23	8	3	3	25	7	2	5	2	2	27	8	3	2

Arthroscopic repair was performed successfully in 36 cases. In two cases, arthroscopic rotator cuff repair was not possible and patients were treated by mini-open repair. In another two cases, a massive rotator cuff tear was encountered, not amenable of surgical repair, so that synovectomy, articular debridement and tenotomy of the long head of the biceps were performed as a symptomatic treatment to reduce pain and postpone reverse arthroplasty surgery.

There were no inter-observer differences in ARoCuS defect categories, and there were no significant differences in the rating of tissue quality (T) between both the observers.

Inter-observer reliability was excellent for all investigated parameters. Except for (T) tissue quality: 0.85 (0.82–0.90), all characteristics showed kappa coefficients of 1. Continuous variables were compared for inter-observer agreement by calculating the interclass correlation coefficient. Δ*V* categories thus showed an ICC of 1.0 (1.0).

Intra-observer reliability was excellent for all investigated parameters as well and showed kappa coefficients of 1. The ICC for Δ*V* was 1.0 (1.0).

Time for calculation of Δ*V* via ARoCuS app was measured in 10 consecutive patients and resulted in a mean time of 25 ± 4 s (starting app to final Δ*V* value). Calculation of Δ*V* without Web application by hand took 2.6 ± 0.5 min and was subject to errors in 40% of the cases.

Postoperative (6 weeks) clinical evaluation of patients resulted in a significant (*p* < 0.001) improved mean UCLA score of 27.8 ± 3.3 compared to the preoperative mean ULCA score of 16.3 ± 2.9.

Five patients (12.5%) that underwent rotator cuff repair developed a post-arthroscopic shoulder stiffness and 2 (5%) had to undergo revision surgery due to a re-tear, possibly due to incompliance to the postoperative protocol. Both tears were previously classified as Δ*V* III, and revision surgery was done as mini-open intervention. There were no complications due to infection (Table 1).

## Discussion

This study demonstrated that the ARoCuS Web app is practicable in use with a fast application time and has an excellent inter- and intra-observer agreement, promoting it therefore as a suitable tool for intraoperative decision-making and standardized documentation of the RC tears.

**Fig. 2:**
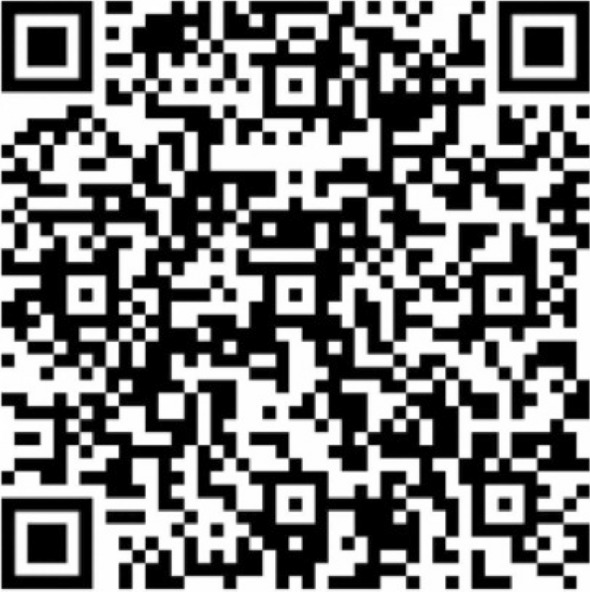
QR code for quick referencing and usage of the ARoCuS app

It is the first study to present a Web-based tool for the calculation, documentation and decoding of the ARoCuS (Fig. 2).

Previously reported high inter-observer reliability of this score was reproduced by a different study cohort and different orthopedic surgeons [11]. However, this study may be somewhat limited by the relatively small number of patients included. Due to high intra- and interrater reliability and the study design without control group, a power analysis was not performed a priori. A post hoc power analysis based on the previously published ICC for inter- and intra-observer reliability found a sample size of 29 individuals sufficient. Furthermore, correlations between preoperative radiological findings and ARoCuS Δ*V* categories were beyond the research scopes of this project. Thus, future studies are encouraged to include more individuals and correlate intraoperative findings to preoperative radiological findings.

So far, the use of ARoCuS has been limited as manual calculation of defect categories Δ*V* was impractical. Through availability of a Web-based app, calculation of defect categories Δ*V* and documentation of intraoperative findings can be integrated into clinical practice. As there is a high inter-observer reliability, the ARoCuS app is suitable as standard tool for intraoperative documentation of rotator cuff tear dimensions. A general and widespread usage of this system could enhance comparability of studies on surgical treatment of rotator cuff tears. Moreover, the integration of this user-friendly Web application into a Web platform for the documentation and scientific exchange of data regarding rotator cuff repair across different hospitals and countries would be a further step to increase the level of evidence in this field of research. Another possible development of this tool is the adaptation and transposition of its structure to a standardized radiological (magnetic resonance arthrography) assessment of rotator cuff tears. This may objectify radiological rotator cuff tear evaluation as current surrogate parameters show high inter-observer differences and may thus be confounding for the actual status of the rotator cuff [15, 16]. Finally, the modular nature of ARoCuS enables it to be extended to describe concomitant pathologies of rotator cuff tears, such as lesions of the capsule, labrum and the long head of the biceps tendon.

Multiple studies describe outcomes after rotator cuff repair, suggesting promising results at short- and long-term follow-up [17–21]. However, comparability of studies and study cohorts describing rotator cuff tears is partially limited as different classification systems and scores have been developed and used in the course of RC tear research [22] and as most of these classifications remain on an ordinal scale [7–9, 23]. Intraoperative evaluation plays a crucial role in RC surgery, not only because it helps selecting the best treatment, but also because it has a predictive role on the success of repair. In fact, recent studies have documented a strong association between RC tear and re-tears at six and nine months after surgery [24, 25]. However, most of the studies which investigated predictive factors for RC re-tear relied on preoperative characteristics, with only few focusing on intraoperative evaluation [26, 27]. Hence, the correlation between anatomical features of RC tears, RC integrity and clinical outcome remains uncertain.

As most intraoperative applied classifications rely on a rather ordinal scale and lack modularity, Web-based applications—to our knowledge—have not been introduced so far in the context of joint surgery. However, rating scales for patient-rated outcome measurements (PROMs) as well as modular clinical assessments of joint functionality have been digitalized and eventually gained widespread acceptance among the clinical and scientific community.

## Conclusions

The ARoCuS Web application is a highly reliable tool that can enhance and simplify the integration of standardized treatment and documentation of rotator cuff tears in clinical routine. This might lead to better comparability of studies reporting on the surgical treatment of rotator cuff tears.
